# A Standardized Evaluation Method for Assessing Patients With Genital Dyschromia

**DOI:** 10.7759/cureus.15840

**Published:** 2021-06-22

**Authors:** Rafael Alves, Tâmara Gomes, Priscilla Baqueiro, Patrícia Fróes Meyer, Daniela Barros, Antonio Schiattarella, Michele Fichera, Laizza Silva, Brenda Ianca de Santana, Patrícia Lordelo

**Affiliations:** 1 Pelvic Floor Care Center, Bahiana School of Medicine and Public Health, Salvador, BRA; 2 Physiotherapy, Potiguar University, Natal, BRA; 3 General and Specialized Surgery for Women and Children, University of Campania Luigi Vanvitelli, Naples, ITA; 4 General Surgery and Medical-Surgical Specialties, University of Catania, Catania, ITA; 5 Medicine, Bahiana School of Medicine and Public Health, Salvador, BRA

**Keywords:** aesthetic gynaecology, uro- gynaecology, clinical evaluation, skin pigmentation, aesthetic dermatology

## Abstract

Objective

The population's ever-growing concern with genital aesthetic dysfunctions reflects an increasing demand in the field of intimate aesthetics. For this reason, as well as the lack of a standardized evaluation, this paper aims to develop a form that facilitates the initial investigation of aesthetic genital dysfunctions.

Methods

An evaluation form for female and male genital dyschromia was developed between July and November 2018. Following initial development, the form was evaluated for quality and was updated by a panel of specialists (a psychologist, two pelvic dysfunction physiotherapists, and two dermato-functional physiotherapists) via email and through a content validity questionnaire. The face validity of the form was assessed by five physiotherapy and medical students who were randomly selected. The students answered a questionnaire evaluating the proposed form. The reliability of the form was established through the test-retest procedure by evaluating its reproducibility over time.

Results

The “Genital Dyschromia Evaluation Form'' (composed of identification, anamnesis, and physical examination sections) was approved by the specialist panel. They suggested questions to be added in the anamnesis (dermatological lesions or fungal and bacterial infections) and physical examination (hyperemia, edema in the perianal and internal thigh region) sections. As for the image analysis, an increase in quality, resolution, and sharpness was suggested. Lastly, for the cutaneous phototype evaluation, the DoctorSkinFototipo® digital analyzer device was chosen since it is small, portable, easily positioned on the genital area, and can be readily cleaned between patients.

Conclusion

The “Genital Dyschromia Evaluation Form” is a questionnaire approved by specialists and could represent a suitable option for health professionals.

## Introduction

The population's concern with genital aesthetic dysfunctions grows continuously. Female intimate aesthetic complaints appear due to new depilatory habits, exposing the genital area more than before. In addition, media and social networks spread the idea of bodily perfection, not just as desirable, but as integral to normative femininity [1-4]. It has been culturally assumed that the vagina has to be tight, small, and all but invisible. Aesthetically, the ideal vagina has been described as a smooth curve, with no visible labia minora [5-6]. Moreover, men are also concerned about intimate aesthetics. Currently, male dissatisfaction is not only restricted to their genital organ size but it may also be associated with its appearance. As a reflection of this new concern, the number of men and women seeking aesthetic treatment for their genital region has grown increasingly [3,7].

Genital dyschromia is an aesthetic disorder that can affect both female and male genital regions and it may develop in the external genitalia or the perianal region [3, 8]. It is a melanin-based pigmentation disorder that may be congenital. Genital hyperchromia can arise due to factors such as aging, hormonal changes, skin friction, obesity, inflammation, allergies, and sun exposure [9]. Hypochromia is rarer in the genital region but it may present be in individuals with vitiligo and it may negatively influence sexual function and quality [10-11].

Genital dyschromia represents one of the most distressing concerns for patients [12-13] and can have a considerable impact on the quality of life and sexual function, like other gynecological conditions which have been widely documented to be associated with psychosocial consequences [14-17].

New research provides therapeutic options for diverse genital aesthetic complaints [8]. These treatments are mostly based on the skin's anatomy and the similarity between the facial tissue and external genitalia. A thorough evaluation is indispensable when choosing the therapeutic option to ensure the success of the treatment [18]. Clinical reasoning during evaluation facilitates the choice of the therapeutic intervention. Some evaluation forms that aim to standardize the evaluation method have already been created. We reviewed the literature and found standardized evaluation charts for aesthetic dysfunctions such as physical therapy evaluation protocol in patients with fibroid edema and physical therapy evaluation protocol for scarring fibrosis levels in the liposuction postoperative period with or without abdominoplasty [19-20]. With the increase in aesthetic complaints in the intimate region and proposals for genital treatments, as well as the lack of a standardized evaluation, a need to create a form that facilitates the initial investigation of aesthetic genital dysfunctions becomes paramount.

Since both female and male genitalia discoloration are frequent aesthetic complaints in clinical practice, this study aims to develop an evaluation form for genital dyschromia.

## Materials and methods

The development of an evaluation form for female and male genital dyschromia occurred between July and November 2018. The study followed the precepts of the Declaration of Helsinki, with approval of the Ethics and Research Committee of the Bahiana School of Medicine and Public Health, under reference number 99473018.2.0000.5544. All participants signed the informed consent form.

Literature research was conducted to identify existing related surveys on the topic. Specialists in intimate aesthetics were contacted and asked to provide any relevant surveys or previously discussed information. 

A workshop was held by local health professionals specialized in intimate aesthetics to identify and finalize criteria, prioritize issues, and define suitable items/scales for inclusion in the form. 

To analyze and improve the evaluation form, five health professionals and researchers from the Pelvic Floor Care Center at the Bahiana School of Medicine and Public Health were consulted and formed an expert panel, as well as applied a content validity questionnaire with each other [21]. The group included a psychologist, two pelvic dysfunction physiotherapists, and two dermato-functional physiotherapists. 

Following the workshops and consultations, the feedback was gathered a draft questionnaire version was generated by the research team.

To check the face validity, five physiotherapy and medical students were randomly selected. The students answered a questionnaire that evaluated the proposed form. The questionnaire was composed of questions to be answered on a three-point Likert scale (good, fair, and poor) and questions requesting yes-or-no answers as follows: questionnaire application date, typographical errors, form length, font size, whether the image was sharp and satisfactory, question and answer options were clear, questions followed a sequence, whether the form's layout was considered to be good, and its final evaluation.

The reliability of the form was established through the test-retest procedure by assessing its reproducibility over time.

## Results

The specialists judged as relevant all the information previously contained in the evaluation form; they contributed suggestions for questions to be added in the anamnesis section and physical examination section of the form. The panelists' suggestions are shown in Table 1.

**Table 1:** Experts panel composed of five professionals.

Nº	Occupational Area	Items to be modified
1	Dermato-functional	Add internal region of thighs in the image; question skin diseases: vitiligo and atopic dermatitis; question about surgeries performed in the perineal region;
2	Dermato-functional	Ask about the presence of vulvovaginitis; during physical examination add the observation of edema and hyperemia;
3	Psychologist	Improve image sharpness; in the main complaint include the patient's perception regarding the dyschromia of the genital region.
4	Urogynecology	Add in the anamnesis the topic of hypochromia; improve image sharpness; include the perianal region for men and women in the image; question the use of MIRENA IUD; during physical examination add the observation of dermatitis and folliculitis;
5	Urogynecology	During physical examination, add the topic of hypochromia; improve image sharpness; include the image of the perianal region for man and woman.

Content validity

As a result of the feedback from the content validity questionnaire, it was determined that questions needed to be added to the anamnesis section: such as the presence of dermatological lesions or fungal and bacterial infections. Similarly, for the physical examination section, it was recommended that hyperemia and edema investigations be added, as well as perianal and internal thigh region examinations. As for the image analysis, improvements in their quality, resolution, and sharpness were suggested. 

Face validity

Of the five students who completed the questionnaires, only one considered the format's layout to be regular and two considered the images to not be sharp enough. The results of the face evaluation questionnaires answers are summarized in Table 2.

**Table 2:** Face validity from the Genital Dyschromia Evaluation Form, answered by five students.

Questions	Good	Fair	Bad
Time of application of form	5	0	0
Acceptable form length	5	0	0
Font size	5	0	0
The form's layout is	4	1	0
Your final evaluation of the form is	5	0	0
Questions	Yes	No	
The image is sharp and reaches the objective	3	2	
The form shows typographical errors	0	5	
The questions are clear	4	1	
The answer options are clear	4	1	
The questions’ order follow good sequence	4	1	

The genital dyschromia evaluation form: final version

The form was denominated as the “Genital Dyschromia Evaluation Form”; the following sections are present in it.

Identification 

It contains questions about the name, address, age, date of birth, occupation, telephone, marital status, education, family income of the pa,tient and by whom the patient was recommended. 

Anamnesis

It starts with questions about the main complaint and is followed by questions about the current disease history, clinical information, and pathological antecedents, gynecological and obstetric history, medicines in use, and life habits.

Physical Examination: To Analyze the Dyschromia and the Cutaneous Phototype

**Figure 1:**
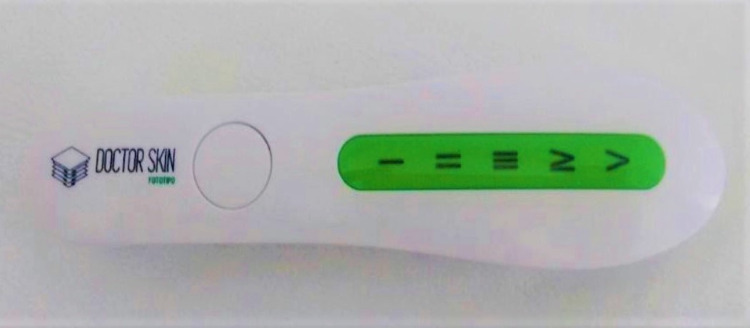
Digital phototype analyzer, DoctorSkinFototipo®.

For the cutaneous phototype evaluation, the digital analyzer device DoctorSkinFototipo® was used since it presented the possibility of use on the genital region (Figure 1). For the cutaneous phototype measurement, the device is pressed against the skin, and the skin tone is identified using five increasing graduations: I being the lightest and V being the darkest skin tone.

To define what intimate region location the equipment should be pressed to the skin, it is necessary to use a disposable ruler so that skin tone measurements before and after therapy are standardized and tested in the same area. To verify skin tone, the patient is positioned in the supine position, with their lower limbs and feet resting on the hospital bed. 

For the cutaneous phototype measurement of the mons veneris and labia majora, the upper margins were selected as the starting point.

**Figure 2:**
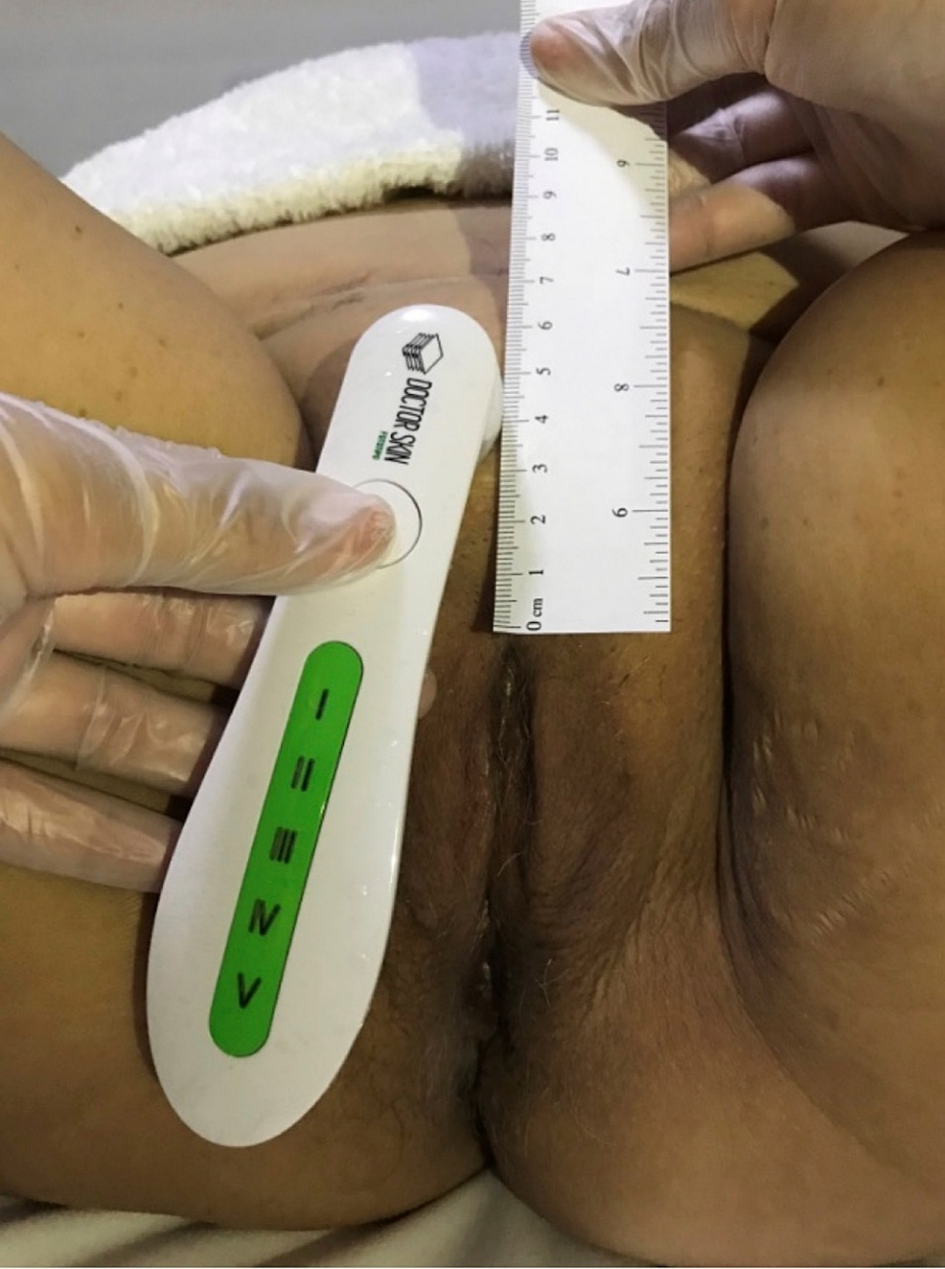
Measurement of cutaneous phototype on the mons Venus, at 4 centimeters above the starting point.

Mons veneris: the ruler's zero mark is placed on the starting point, and the measurement is made at a distance of 2 to 6 centimeters above the zero mark (Figure 2), as well as from 2 to 6 centimeters to the right and left sides. 

Labia Majora: the ruler's zero mark is placed on the starting point, and the measurement is made at 2 to 6 centimeters below the zero mark, bilaterally. 

For the cutaneous phototype inner thigh measurement, the zero mark will become the labia majora's midpoint, measured with the disposable ruler.

Inner Thigh: the ruler's zero mark is placed on the starting point and the measurement is made at 2 to 6 centimeters sideways to the labia majora. 

**Figure 3:**
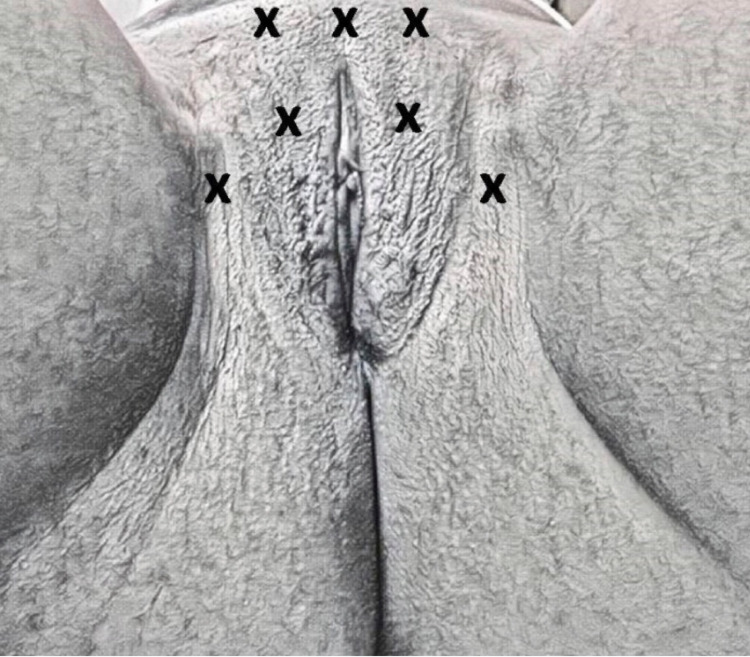
Sample area measurements of cutaneous phototype on the external genitalia region.

To ensure the phototype analysis consistent results, it should always be performed in the same location, each evaluation requires the therapist to identify in the evaluation form how many centimeters each region was analyzed from. Figure 3 illustrates the cutaneous phototype measurement points on the external genitalia.

The weight and height of the patient as well as genital region verification for altered tactile sensitivity were also added to the evaluation form. A Wood lamp was used to exclude fungal hyperpigmentation.

## Discussion

This evaluation form was created to assist specialists working on female and male genital dyschromia. It is believed the "Genital Dyschromia Evaluation Form" will be of great value in the formulation of a therapeutic proposal. During the process of the form building, all panel expert professionals' contributions, content validity were relevant and included in the validation proposal. This was a significant step in the creation process taking into consideration the specialist's opinion being fundamental for all related aspects to the subject to be contemplated, as well as confirming the demand for interaction from a multidisciplinary and interdisciplinary team [22].

The use of the skin phototype analyzer DoctorSkinFototipo® in the evaluation form was accepted and praised by the specialist professionals, as it brings in an innovative method that provides a more objective assessment of the skin phototype, without depending exclusively on the examiner's expertise. It is believed that this new device may even serve as a parameter for evaluation and comparison before and after treatments for skin tone uniformity in the genital region. The photo analyzer device tool was chosen since it was small, portable, readily positioned on the genital area, and easily cleaned between patients. The use of the disposable stretch film fully prevents infectious bacterial transmission and must be placed on the photo analyzer device tool between the part that contacts the patient and the patient's skin during the examination.

It is also worth mentioning as part of a health professional’s routine, the use of personal protective equipment in clinical dermatology practice includes hand hygiene, latex examination gloves, environmental disinfection, which configure important components to protect patients from cross‐infection. 

The individual's body perception was considered a relevant criterion for identifying the relationship between this individual and his/her aesthetic dysfunction as well as the acknowledgment of the main complaint being in alignment with what is observed by the evaluator. Body image evaluation and genital image perceptions represent an important criterion for sexual health and must be included in aesthetic dysfunction evaluations [23]. As a way of recognizing genitalia's color, the lighter skin tone shade which corresponds to a hypochromia was inserted in the evaluation form. Similar to facial and body regions, the genital region may have lighter tones patches due to tissue dehydration, dysfunction in the melanogenesis process, or destruction of melanocytes, as is the case with vitiligo [9-10]. 

According to the specialist's panel suggestions following content validity, the presence of dermatological pathologies in the genital region and possible alterations in the genital flora such as vulvovaginal candidiasis and bacterial vaginosis were questions inserted in the evaluation form. The lack of tissue integrity or the presence of gynecological pathologies may limit the treatment of intimate aesthetics since they are contraindicated criteria for the use of therapeutic resources [24]. In addition, atopic dermatitis can extend into the genital region and become an important warning to the use of products and active principles as a form of treatment.

Specialists were concerned about any previous surgery performed in the perineal region and if a contraceptive method was being used were included in the evaluation form's gynecological history. Previous surgery in the perineal region was considered a relevant factor since a surgical submission condition can trigger altered scars and generate discomfort. Regarding the use of the IUD as a contraceptive method, the different types (copper or Mirena®) were added as question items in the evaluation form, since it is known that different mechanisms actions in the organism may cause different hormonal actions [25-27]. 

In the physical examination, suggestions to be modified by the specialists through the panel and content validity as described: the presence of edema and hyperemia on the vulva, replace the current image for its lack of good resolution and include perianal and internal thigh region evaluations. Hyperemia and edema signs were relevant because they may be associated with gynecological or skin pathologies [24]. In the face validity analysis evaluation, it was also reported a deficiency in image sharpness. Initially, the software used to create the image left it darker and unclear, the image was then modified based on the standardization of the genital images described by Joumblat et al. [28]. During the physical examination, the perianal region and the upper third of the internal thigh were added, since the intimate aesthetics dysfunction may extend to these regions [24]. Coloration increase in the internal thigh can occur due to the increase of local adipose tissue and consequent friction [9]. 

The evaluation form update was done from the clinical practice, selecting what items approach best the signs and symptoms presented by patients. It is suggested the evaluation form be applied in patients who present genital dyschromia complaints. 

This research was carried out in a single reference center and face validity was composed only by undergraduate students. For future perspectives, it is hoped this validation can be gathered from different populations.

## Conclusions

The “Genital Dyschromia Evaluation Form” could represent a suitable option for health professionals. A standardized and dedicated evaluation method for diagnosing patients with genital dyschromia makes it simple to gather reliable data with adequate construct validity for use by health professionals.

## Figures and Tables

**Figure 4:**
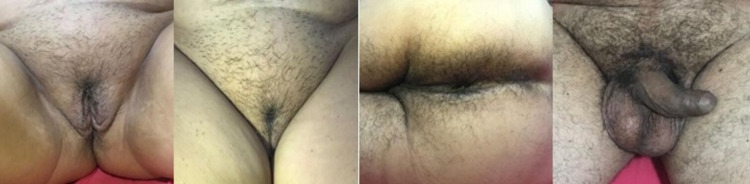
Circle the spot area. (Images belonging to the author, with permission for publication)

**Figure 5:**
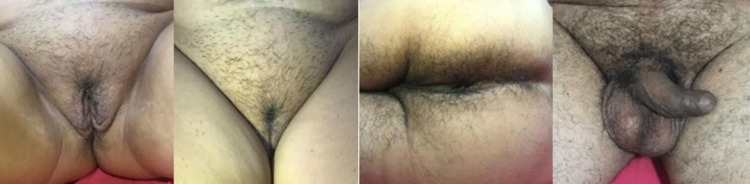
Circle the dyschromia'a area Images belonging to the author, with permission for publication

## Appendices

Genital Dyschromia Evaluation Form

Identification

Name: ___________________________      Gender: (   ) Female (  ) Male

Address: _________________________

Age: _____   Date of birth: ____/____/______   Telephone: ___________

Profession: ________________  Occupation: ______________________

Marital status:  (   ) single   (   ) married   (   ) divorced  (   ) widowed

Education:  (   ) Elementary  (   ) High school   (   ) College  (   ) University

Income (number of minimum wages): (   ) 0- 3  (   ) 4- 6  (   ) 7-9  (   ) ≥ than 10

Anamnesis

What is your perception regarding the coloration of your genital region?

Spot type: Light (   ) Dark (   ) Both (   )

Spot color: (   ) Bluish  (   ) Grayish (  ) Browned (   ) Blackened  (  ) Hypochromia (whitish)

Location:  (   ) Spot (   ) Generalized

Circle the spot area in Figure 4.

Please check the pigmentation area: (   ) Labia majora (   ) Labia minora (   ) Groin (   ) Intergluteal area  (   ) Perianal region (   ) Inner thigh 

Do these spots make you feel uncomfortable with your partner?  (   ) No (   ) Yes

If yes, rate from 0 to 10 your level of embarrassment:

Do you have a regular partner?  (   ) No (   ) Yes 

If answered yes, have you and your partner talked about your discomfort regarding your genital area?  (   ) No (   ) Yes

If yes for the question above, does he (she) agree with your complaint?  (   ) No (   ) Yes 

Is he/she aware you are seeking treatment for your complaint?  (   ) No (   ) Yes 

Do you feel embarrassed to expose your genitals to either friends, family, or health care professionals due to your spot?  (   ) No (   ) Yes 

If answered yes, specify who: (   ) Partner (   ) Friend (   ) Family (   ) Health care professional

Does your dyschromia limit you from having any kind of social activity?  (   ) No (   ) Yes 

If so, which? 

Does your dyschromia limit your sexual life?  (   ) No (   ) Yes 

Do you often look at your genitalia?  (   ) No (   ) Yes. If so, how frequently? 

Current Disease History

Dyschromia emergence:

How long ago has your dyschromia first appeared? 

(in years) (  ) < 1 (  ) 1- 2  (  ) 3- 4  (  ) ≥ 10

Please check the initial position of where your dyschromia first appeared: 

(  ) Obesity/Localized Fat 

(  ) Friction/Tight clothing 

(  ) Hair removal  blade  

(  ) Hair removal wax

(  ) Hair removal Laser

(  ) Hair removal  Pulsed light

Please check whether you associate the emergence of your dyschromia with any of the below:

(  ) Contraceptive 

(  ) Menopause 

(  ) Endocrine disruptors

(  ) Pregnancy

(  ) Physical activity 

(  ) Other __________

Have you ever undergone any previous treatment? (   ) No (   ) Yes 

If so, what kind? (   ) Cosmetic (   ) Chemical peeling (   ) Phototherapy  (   ) Other ___________________

How long ago? ______________ 

Please rate your satisfaction level to prior treatments (0-10): 

Clinical Information and Pathological History

Please mark below for thyroid disorders: 

Hypothyroidism (   ) Yes (   ) No    Hyperthyroidism  (   ) Yes (   ) No    Absence of thyroid  (   ) Yes (   ) No  

Have you been diagnosed with Vitiligo?  (   ) Yes (   ) No  

Have you been diagnosed with atopic dermatitis?  (   ) Yes (   ) No  

Do you currently have any vulvovaginitis (vaginal candidiasis or bacterial vaginosis)? (   ) Yes (   ) No  

Do you currently have any dermatological conditions in the genital area? (   ) Yes (   ) No.  

If so, please mark one of the options below:

(   ) Dermatitis  (  ) Allergies (   ) Folliculitis  (   ) Pseudofoiliculitis  (   ) Herpes (   )  Others _______________

Do you have facial melasma: (   ) Yes (   ) No

Do you have any hepatic condition? (   ) Yes (   ) No

Gynecological and Obstetric History

Age of first menstrual period _______ Date of last menstrual period ______/______/_____

Children: Number of pregnancies ___ Cesarean ___ Natural Childbirth ____ Abortion(s)____

Are you currently breastfeeding? (   ) Yes (   ) No

Are you sexually active? (   ) Yes (   ) No. If so, how many times per week? (  )< 2 x  (  ) 2-4 x  (  ) ≥ 5x

Are you currently in menopause (   ) Yes (   ) No. If so, for how long?  

Have you had any surgeries in the perineal region? (   ) Yes (   ) No

Medications in use 

Which contraceptive method are you currently using?

(   ) Oral contraceptive pill  (   ) Hormonal injectable contraceptive (   ) Skin adhesives with hormones (   ) Hormonal implants  (   ) Vaginal ring

(   ) Copper IUD  (   ) Mirena® IUD (    ) None

Are you currently undertaking any hormone therapy?  (   ) Yes  (   ) No. If so, what kind? _________________

Are you currently taking any other medications? (   ) Yes  (   ) No. If so, what kind? ____________________

Life Habits

Are you currently practicing any physical activity? (   ) Yes (   ) No. If so, what kind and how frequent? ______________________________

Do you smoke? (   ) Yes (   ) No. If so, how many packs per day? (   ) 1 (   ) < 2  (   ) 3 - 4 (   ) > 5

Do you consume alcohol? (   ) Yes (   ) No. If so, how much alcohol do you drink per week? (   ) 1 (   ) < 3  (   ) 3 - 4  (   ) > 5

Do you expose your genital region to the sun? (   ) Yes (   ) No. If so, how often per week? (   ) 1 day (   ) < 3  (   ) 3 - 4  (  ) > 5

What types of underwear fabric do you most frequently wear? (   ) Cotton (   ) Lycra. Does it have visible stitching? (   ) Yes (   ) No

Do you wear tight clothes? (   ) Yes (   ) No. If so, how often per week? (   ) 1 day (   ) < 3  (   ) 3 - 4  (   ) > 5

What is your genital area hair removal method: (  ) Blade   (  ) Hot wax  (  ) Cold wax (   ) Laser   (   ) Pulsed light 

How frequently do you remove your pubic hair? (  ) 1x per week (  ) < 3  (  ) 3 - 4  (  ) > 5  or (   ) Once per month  (   ) ≥ Every 2 months 

Physical Examination (to be completed by the health professional)

Skin appearance and state: (   ) Hydrated (   ) Dehydrated   

Skin condition: (   ) Flaccidity (   ) Genital furrows and wrinkles (   ) Dermatitis (   ) Folliculitis

Vulva appearance: (   ) Swollen (   ) Hyperemesis (   ) Does not apply

Circle the dyschromia's area in Figure 5:

Closest spot tonality: (  ) Bluish (   ) Grayish (   ) Browned (   ) Blackened (   ) Hypochromia

Post-inflammatory spots: (   ) Yes (   ) No.  If so, where? 

Cutaneous phototype

Venus mound (female) or pubis (male) : _____ centimeters (cm) from starting point

           (   ) I                 (   ) II                      (   ) III                       (   ) IV                        (   ) V

Labia majora (female) or base of the penis and scrotum: _____ cm from starting point (distinguish right and left side)

           (   ) I                 (   ) II                      (   ) III                       (   ) IV                        (   ) V

Inner thigh: _____ cm from starting point

           (   ) I                 (   ) II                      (   ) III                       (   ) IV                        (   ) V

Patient's weight: _________    Patient's height: _________     

Changes in skin sensitivity: (   ) Yes (   ) No. Where: 

Wood's Lamp examination: (   ) Epidermal  (   ) Dermal
